# Directional auxin fluxes in plants by intramolecular domain–domain coevolution of PIN auxin transporters

**DOI:** 10.1111/nph.16629

**Published:** 2020-05-20

**Authors:** Yuzhou Zhang, Corinna Hartinger, Xiaojuan Wang, Jiří Friml

**Affiliations:** ^1^ Institute of Science and Technology (IST) Austria Klosterneuburg 3400 Austria; ^2^ College of Life Sciences Northwest University Xi’an 710069 China

**Keywords:** cellular polarity, directional auxin flow, domain swapping, domain–domain coevolution, PIN auxin transporters, subcellular membrane localization

## Abstract

- Morphogenesis and adaptive tropic growth in plants depend on gradients of the phytohormone auxin, mediated by the membrane‐based PIN‐FORMED (PIN) auxin transporters. PINs localize to a particular side of the plasma membrane (PM) or to the endoplasmic reticulum (ER) to directionally transport auxin and maintain intercellular and intracellular auxin homeostasis, respectively. However, the molecular cues that confer their diverse cellular localizations remain largely unknown.
- In this study, we systematically swapped the domains between ER‐ and PM‐localized PIN proteins, as well as between apical and basal PM‐localized PINs from *Arabidopsis thaliana*, to shed light on why PIN family members with similar topological structures reside at different membrane compartments within cells.
- Our results show that not only do the N‐ and C‐terminal transmembrane domains (TMDs) and central hydrophilic loop contribute to their differential subcellular localizations and cellular polarity, but that the pairwise‐matched N‐ and C‐terminal TMDs resulting from intramolecular domain–domain coevolution are also crucial for their divergent patterns of localization.
- These findings illustrate the complexity of the evolutionary path of PIN proteins in acquiring their plethora of developmental functions and adaptive growth in plants.

## Introduction

The plant hormone auxin and its graded distribution between cells govern almost all aspects of plant growth and development (Benková *et al*., 2003; Gray, 2004; Vanneste & Friml, 2009; Fendrych *et al*., 2016; Lavy & Estelle, 2016; Robert *et al*., 2018), as well as plant adaptive growth in response to environmental cues (Ding *et al*., 2011; Su *et al*., 2017; Grones *et al*., 2018; Rakusová *et al*., 2019). Unlike other phytohormones, auxin‐dependent plant morphogenesis and adaptabilities rely on auxin concentration gradients that are, besides the contribution of local auxin biosynthesis (Brumos *et al*., 2018), established by an active, directional cell‐to‐cell polar auxin transport mediated by auxin transporters, among which, the PIN‐FORMED (PIN) auxin exporters play a plethora of developmental roles (Swarup *et al*., 2001; Blakeslee *et al*., 2005; Adamowski & Friml, 2015; Park *et al*., 2017). PINs belong to a class of transmembrane proteins and display polar localization at the membrane to mediate directionality of auxin flow (Petrášek *et al*., 2006; Wisniewska *et al*., 2006; Tejos *et al*., 2014; Langowski *et al*., 2016; Nodzyński *et al*., 2016).

Previous analyses of PIN protein structure have identified a tripartite domain structure that includes two predicted transmembrane domains (TMDs) at both the N‐ and C‐termini and a central hydrophilic loop (HL) (Křeček *et al*., 2009). Compared to the HL, the TMDs are relatively evolutionarily conserved among PIN family members (Bennett *et al*., 2014). From an evolutionary perspective, *PIN* genes have been found in almost all land plant lineages (Viaene *et al*., 2013, 2014; Bennett, 2015), in agreement with the presence of auxin signaling components and auxin responses in land plants (Mutte *et al*., 2018). Recently, an ancestral form of *PIN* was identified in the green alga *Klebsormidium*, which shows plasma membrane (PM) localization in algal cells and was verified to transport auxin molecules as well (Skokan *et al*., 2019). In terms of phylogenetic relationships and subcellular localization, the PIN protein family members are generally grouped into three types: the canonical, PM‐localized PINs that mediates cell‐to‐cell auxin flow for intercellular auxin homeostasis; the noncanonical, endoplasmic reticulum (ER)‐localized PINs that presumably mediate auxin exchange between the cytosol and ER‐lumen possibly contributing to intracellular auxin homeostasis; and dual PM‐ and ER‐localized PINs such as *Arabidopsis* PIN6 with unclear function (Mravec *et al*., 2009; Ding *et al*., 2012; Cazzonelli *et al*., 2013; Bennett *et al*., 2014; Ganguly *et al*., 2014; Verna *et al*., 2015; Simon *et al*., 2016). Additionally, the canonical PINs are polarly distributed at the PM of specific cells to guide directional auxin flow (Wisniewska *et al*., 2006; Glanc *et al*., 2018). For instance, in the model flowering plant *Arabidopsis*, four of five canonical PINs, PIN1/3/4/7, are localized to the basal side of root stele cells to mediate the formation of an auxin maximum in root tips that is recognized as the pattern‐ or organ‐organized signal (Friml *et al*., 2002; Billou *et al*., 2005), while PIN2 is apically localized in root epidermal cells to exclusively transport auxin from the root tip to the elongation zone to mediate root gravitropic growth (Müller *et al*., 1998; Baster *et al*., 2013; Zhang *et al*., 2019).

Despite great advances in our understanding of the crucial role of PIN proteins at the different subcellular destinations for plant development and adaptive growth, we still have limited knowledge of the molecular cues that determine these divergent localizations of PIN family members. Uncovering the mysteries of sequence‐dependent diversification of the subcellular and polar localization of PIN proteins will not only reveal the evolutionary processes that underlie the establishment of divergent developmental roles of PIN proteins, but will also open a window into a better understanding of the evolution of higher plants with their richness of forms and multifaceted growth adaptability to different environmental conditions.

## Materials and Methods

### Plant materials and growth conditions

The loss‐of‐function mutant *pin2* was previously described (Müller *et al*., 1998). *Arabidopsis thaliana* seedlings were grown vertically in Petri dishes on ½ Murashige & Skoog (MS) medium (pH 5.9) containing 1% sucrose and 0.8% agar at 18°C under a long‐day light regime (light intensity: 250 µmol m^–2^ s^–1^). The vertical growth index (VGI) of *Arabidopsis* roots were measured as previously described (Grabov *et al*., 2005). The parameters for the calculation of VGI were measured using imagej software on > 10 seedlings per genotype (NIH; http://rsb.info.nih.gov/ij).

### Vector construction and plant transformation

To construct the PIN‐GFP (green fluorescent protein) fusion proteins, the *GFP* gene was fused in‐frame to the central HL domain of PIN protein open reading frames by performing overlapping PCR. To construct the chimerical PIN proteins containing combinations of TMDs and HLs from different PIN family members, the central HL and TMD from the N‐ or C‐termini were cloned individually, after which we performed overlapping PCR to fuse the three fragments together. Following this, the *PIN‐GFP* and chimeric *PIN* PCR products were individually cloned into the Gateway entry vector pDONR221 by the BP reaction. A 1.4 kb fragment containing the *PIN2* promoter was cloned into the Gateway entry vector pPONRP4P1r, and the two were then fused and cloned into the Gateway destination vector pB7m24GW.3 by the LR reaction. The primers used to generate these constructs are shown in Supporting Information Table S1. Transgenic *Arabidopsis* plants were generated using the floral dip method and selected on solid, 0.5× MS medium containing 15 μg ml^−1^ of Basta herbicide (glufosinate). To confirm successful plant transformation, following herbicide selection, seedlings carrying the GFP fusion proteins were further selected by examination under the fluorescence microscope.

### Confocal microscopy

To analyze the polarity and subcellular localization of proteins in the transgenic lines described above carrying the GFP‐PIN fusion proteins, the plants were separately crossed with *pPIN2::PIN2‐mCherry* and the ER marker line *35S::HDEL‐RFP* (Liu *et al*., 2011). We then observed the GFP, mCherry and RFP (red fluorescent protein) signals in 5‐d‐old *Arabidopsis* root tips using a Zeiss 800 inverted confocal laser scanning microscope.

### Identification of coevolving sites

The PIN protein sequences used for the coevolution analysis were retrieved from Phytozome (https://phytozome.jgi.doe.gov/pz/portal.html#) in 15 plant species (Dataset S1), including *A. thaliana*, *Populus trichocarpa*, *Capsella rubella*, *Brassica oleracea capitata*, *Carica papaya*, *Medicago truncatula*, *Crocus sativus*, *Gossypium raimondii*, *Glycine max*, *Vitis vinifera*, *Sorghum bicolor*, *Brachypodium distachyon*, *Oryza sativa*, *Zea mays* and *Ananus comosus*. Identification of pairs of coevolving sites in the correlated regions of N‐ and C‐terminal TMDs of PIN proteins was achieved by running the spidermonkey/BGM program (Poon *et al*., 2008), which was implemented in the hyphy software package (Kosakovsky Pond *et al*., 2005). This reconstructs the substitution history of the alignment by maximum likelihood‐based phylogenetic methods, and then analyzes the joint distribution of substitution events using Bayesian graphical models to identify significant associations among sites. We set the minimum number of nonsynonymous substitutions per codon site to 1, and the maximum number of parents per node to 2. The PIN amino‐acid sequences are run under default settings (a 10^4^ ‘burn‐in’ followed by 10^5^ steps thinned to 100). A posterior probability value ≥ 0.5 was used as the definition of association between coevolving sites.

## Results

### Sequence‐based determinants of *Arabidopsis* PIN protein subcellular localizations

**Fig. 1:**
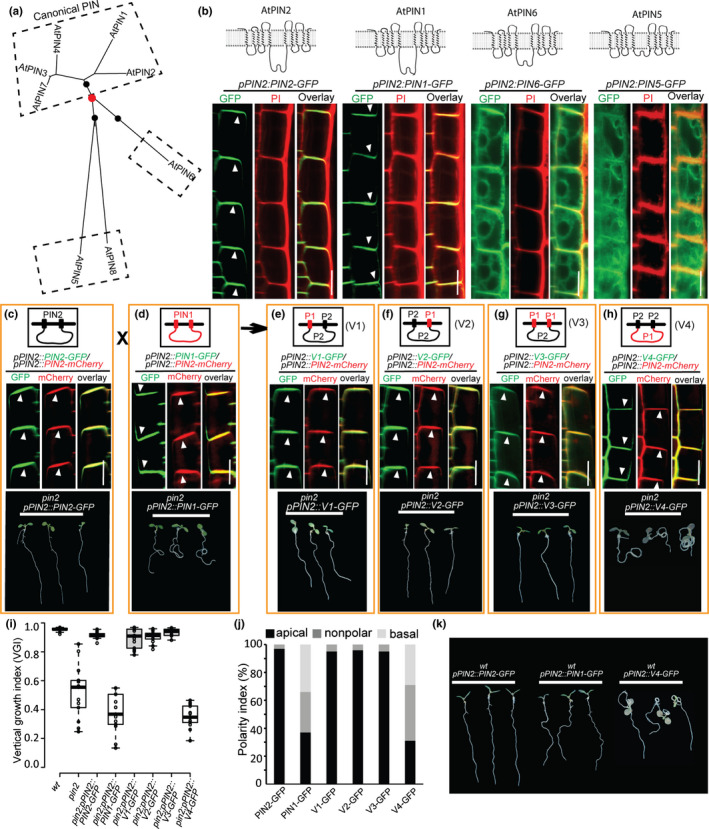
The divergent hydrophilic loop (HL) domains confer the polarity of canonical PIN. (a) Phylogenetic analysis of the eight *Arabidopsis* PIN gene proteins based on their sequence similarities and subcellular localization. The unrooted tree is divided into three clades as indicated by the three black dots. (b) Subcellular localization analysis of four PIN proteins from three clades in *Arabidopsis* root epidermal cells: *PIN* genes tagged with GFP were expressed under control of the *PIN2* gene promoter. PIN1 and PIN2 represent the canonical PIN clade, while PIN6 and PIN5 represent the other two noncanonical clades, respectively. (c) Coexpression of PIN2‐GFP with PIN2‐mCherry showed their intimate colocalization to the apical side of the root epidermal cells (middle panel), and this apically localized PIN2‐GFP successfully rescued the defective root gravitropism phenotype of the *pin2* mutant (bottom panel). (d) the PIN1‐GFP fusion protein shows nonpolar localization in root epidermal cells as indicated by its uncoupled colocalization with PIN2‐mCherry (middle panel), and it also failed to rescue the *pin2* mutant phenotype (bottom panel). (e–h) Top panels: schemes for the intragenic domain swapping experiments between PIN1 and PIN2. These chimeric PIN proteins are composed of the transmembrane domains (TMDs) at the N‐ and C‐termini and a central HL from PIN1 or PIN2. Middle panels: coexpression analyses of these chimeric PIN proteins with apically PM‐localized PIN2‐mCherry; V1 has the N‐TMD of PIN1 and the C‐TMD/HL of PIN2 (e), V2 has the C‐TMD of PIN1 and the N‐TMD/HL of PIN2 (f), and V3 has the C‐ and N‐TMDs of PIN1 and the HL of PIN2 (g). The nonpolar localization of V4, which consists of the C‐ and N‐TMDs of PIN2 and the HL of PIN1, is shown in (h). All experiments were conducted in *Arabidopsis* root epidermal cells. Bottom panels: genetic complementation experiments show that only the apically localized PIN proteins (PIN2, V1, V2 and V3) complement the defective gravitropism phenotype in the *pin2* mutant (c, e–g). (i) Quantification of the vertical growth index (VGI, as explained in Supporting Information Fig. S2b) of the transgenic *Arabidopsis* lines. Center lines show the medians; box limits indicate the 25^th^ and 75^th^ percentiles as determined by R software; whiskers extend 1.5 times the interquartile range from the 25^th^ and 75^th^ percentiles, and outliers are represented by dots. (j) Polarity index of the PIN protein localization in the root epidermal cells (*n* = 100 cells from 10 roots). (k) In contrast to the apically localized PIN2, expression of the PIN1 and V4 proteins in the wild‐type background under control of the *PIN2* promoter may antagonize the endogenous PIN2 function and lead to the agravitropic phenotype. The white arrowheads (b–h) indicate the direction that the PIN proteins localize to. Scale bars, 10 µm.

In *Arabidopsis*, there are eight PIN protein family members. In terms of sequence similarity and the length of the central HL, they are grouped into the following three evolutionary clades (Bennett *et al*., 2014; Adamowski & Friml, 2015): the canonical PINs (PIN1, PIN2, PIN3, PIN4 and PIN7) with long HLs (> 350 residues); two noncanonical PINs (PIN5 and PIN8) that have short HLs (< 50 residues); and another noncanonical PIN (PIN6) with an HL of intermediate length (> 250 residues) (Fig. 1a). To study the properties of their sequence‐dependent subcellular localization, we used the *Arabidopsis* root epidermal cell as the unified cell type and expressed all these PIN proteins under control of the *PIN2* promoter (Fig. 1b).

PIN1 and PIN2 are localized to the PM of root epidermal cells (Fig. 1b–d). However, unlike the exclusively apically localized PIN2 protein, PIN1 localizes predominantly to the basal side of epidermal cells as previously reported (Wisniewska *et al*., 2006; Feraru *et al*., 2011). Consistent with previous findings (Simon *et al*., 2016), the noncanonical PIN, PIN6, simultaneously shows PM and ER subcellular localization in root epidermal cells, while the noncanonical PIN5 is predominantly localized to the ER (Fig. S1). Moreover, compared to the meristematic zones, PIN6 showed enhanced ER localization in the transition zone.

These experiments confirm a different subcellular localization of different clades of PIN proteins and clarify that compared with cell‐type‐specific determinants, these diverse localizations are based on sequence‐specific signals.

### Role of PIN hydrophilic loop in determination of canonical PIN polarity at the PM

As shown above, the canonical PINs, PIN1 and PIN2, localize to the PM in root epidermal cells, but they show differences with respect to the polarity of their localization. Specifically, PIN2 is apically localized in root epidermal cells, as revealed by anti‐PIN2 immunolocalization (Müller *et al*., 1998) and by tagging with GFP or mCherry (Figs 1c, S2a). PIN1, however, shows a basal or nonpolar localization pattern demonstrated by the failure of PIN1‐GFP to be intimately colocalized with apically localized PIN2‐mCherry in epidermal cells (Figs 1d, S2b). We then wanted to know which topological structural parts of PIN proteins confer the molecular signals that determine this differential polarization in the PM.

Intragenic domain swapping experiments revealed that when either the N‐ or C‐terminal TMD of PIN2 was replaced with the N‐ or C‐terminal TMD from PIN1, the two chimeric PIN proteins (named V1 and V2, respectively) still showed the predominantly apical cellular localization in epidermal cells, as verified by their colocalization with PIN2‐mCherry (Figs 1e,f, S2c,d). Additionally, when both the N‐ and the C‐terminal TMDs of PIN2 were simultaneously replaced with the N‐ and C‐ TMDs from PIN1, this chimeric PIN protein (V3) still showed apical localization in the PM, again confirmed by its colocalization with PIN2‐mCherry (Figs 1g, S2e). Together, these results suggest that for the canonical PINs, the HL rather than the TMDs probably underwent evolutionary selection and thus determines their polar localization in the PM. To verify this hypothesis further, we replaced the central HL of PIN2 with that of PIN1, and this chimeric PIN (V4) indeed lost the ability to localize to the apical side of the epidermal cells, as indicated by its uncoupled colocalization with PIN2‐mCherry (Figs 1h,j, S2f). Moreover, besides in the root epidermal cells, we also analyzed the cellular localizations of these PIN proteins and chimeras in cells of the lateral root cap (LRC) and cortex under control of the *PIN2* promoter (Fig. S3). Consistent with their polarity analysis in root epidermal cells, the PIN1 and chimeric protein V4 failed to be apically localized in LRC cells, while the PIN2 and chimeras V1–V3 with the HL of PIN2 were localized to the apical side in this cell type (Fig. S3). These findings revealed a crucial role of the HL in the polarity of canonical PIN localization. Notably, both PIN1 and PIN2 showed a basal localization in cortical cells as previously reported (Wisniewska *et al*., 2006), and all chimeric PIN proteins V1–V4 were basally localized as well (Fig. S3). This indicates that cell type also contributes to the determination of PIN polarity.

The localization of PIN proteins on the basal vs apical sides of the cells is tightly linked to their physiological functions in plant adaptive growth (Glanc *et al*., 2018). To analyze the functions of these chimeric PIN proteins, we introduced them into the *pin2* mutant under control of the *PIN2* promoter. The PIN2‐GFP fusion protein was able to restore the *pin2* defect in root gravitropism (Figs 1c, S4), whereas PIN1‐GFP failed to rescue this defect (Fig. 1d). The apically localized chimeric PIN proteins V1, V2 and V3 nicely complemented the *pin2* mutant phenotype (Fig. 1e–g), whereas the basally PM‐localized chimeric PIN (V4) failed to complement the *pin2* mutant gravitropic growth defect (Fig. 1h,i).

Moreover, in contrast to PIN2‐GFP, expression of the PIN1‐GFP fusion protein or the chimeric PIN (V4) protein under control of the *PIN2* promoter in the wild‐type background resulted in agravitropic root growth (Fig. 1k), suggesting that the basally PM‐localized PIN1‐GFP and V4‐GFP fusion proteins may interfere with apical (shootward) auxin flow directed by apically localized endogenous PIN2 protein and therefore interfere with root gravitropism.

These results are congruent with the cellular polar localization of these chimeric PIN proteins and confirm the crucial role of the apical PIN2 localization in its function for root gravitropism.

### Role of hydrophilic loop in determining PM vs ER localization of PIN proteins

Compared with the PM‐localized canonical PIN, the noncanonical PIN6, which possesses an HL of intermediate length, displays a dual localization in both the PM and the ER membrane (Fig. 1b). To reveal which topological structural part between PIN2 and PIN6 leads to their divergent subcellular localization, we systematically constructed various hybrid PIN proteins by domain swapping.

**Fig. 2:**
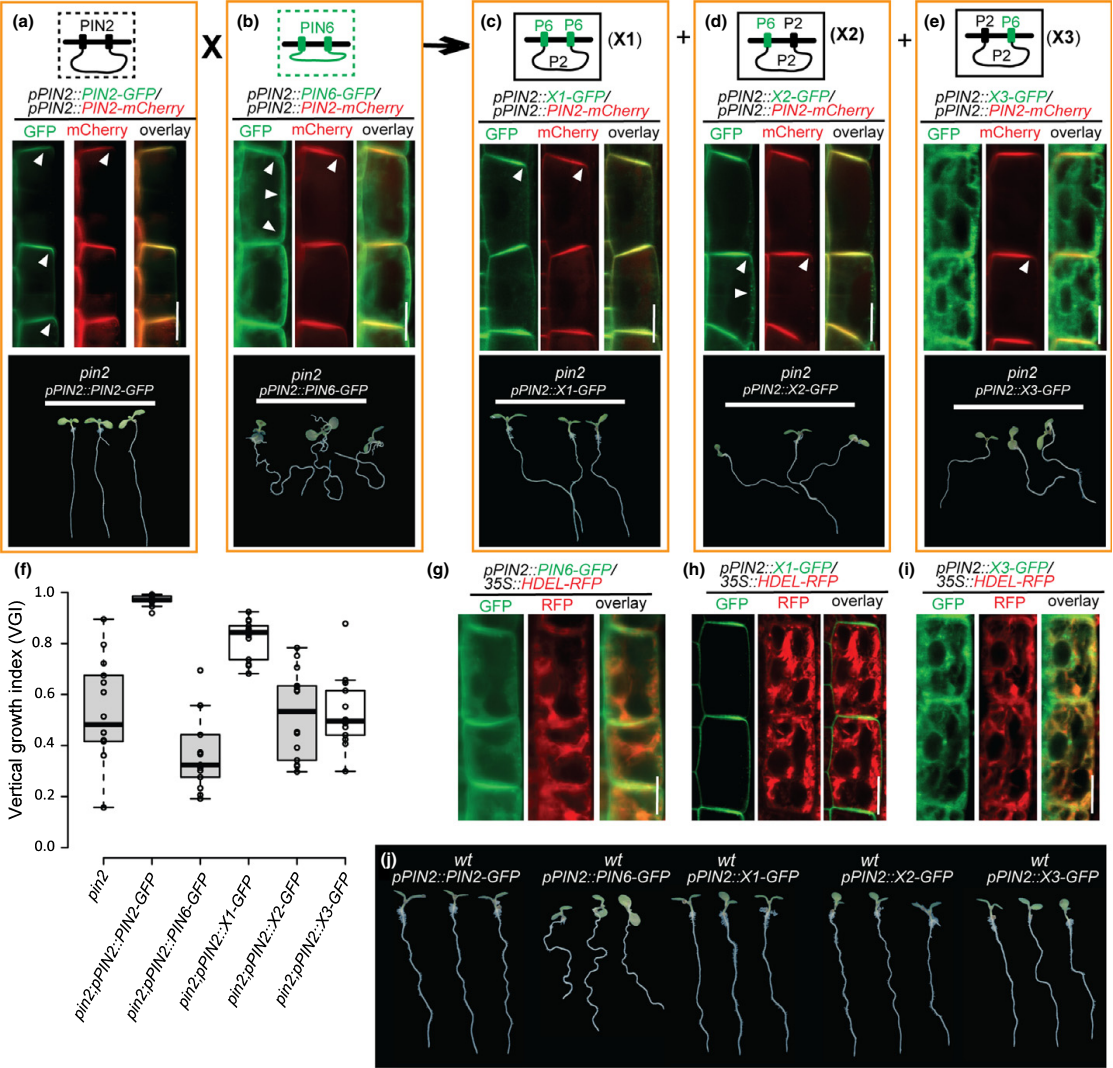
The sequence‐divergent hydrophilic loop (HL) domains define the subcellular localization of PIN proteins to the PM or ER. (a–e) Top panels: diagrams of the structures of PIN2, PIN6 and three chimeric PINs (X1, X2 and X3) with the domains swapped between the canonical PIN (PIN2) and noncanonical PIN (PIN6). Middle panels: analyses of the subcellular localization of PIN2, PIN6 and the chimeric PINs by coexpressing their GFP fusion proteins with the apically PM‐localized PIN2‐mCherry fusion protein. PIN6 shows dual localization at both the PM and the ER (b), while the chimeric X1 protein with the C‐ and N‐TMDs from PIN6 and the HL domain from PIN2 shows PM localization with apical polarity (c). The X2 protein with N‐TMD from PIN6 and N‐TMD/HL domains from PIN2 is localized to the PM (d), but the X3 chimera, with the PIN6 C‐TMD and the PIN2 N‐TMD and HL, is predominantly localized to the ER (e). Bottom panels: results of genetic complementation experiments indicating that compared with PIN2 (a), the apical PM‐localized X1‐GFP protein is able to partially rescue the *pin2* defect in root gravitropism (c), whereas the PIN6, X2 and X3 proteins failed to do so (b, d and e). The white arrowheads (a–e) indicate the direction that the PIN proteins localize to. (f) Quantification of the vertical growth index (VGI) of the transgenic lines (a–e). Center lines show the medians; box limits indicate the 25^th^ and 75^th^ percentiles as determined by R software; whiskers extend 1.5 times the interquartile range from the 25^th^ and 75^th^ percentiles, and outliers are represented by dots. (g–i) Colocalization analysis of the GFP fusion proteins PIN6, X1 and X3 along with the ER marker HDEL‐RFP. (j) Root gravitropic phenotypes of *PIN* gene transformants in the wild‐type *Arabidopsis* background. Expression of PIN6‐GFP driven by the *PIN2* promoter results in defective root gravitropism, consistent with the nonpolar localization of the PIN6 protein (b). Scale bars, 10 µm.

First, we replaced the HL of PIN6 with the PIN2‐HL and called this chimeric PIN protein X1. When compared with PIN6, the X1 chimera displayed the predominant PM localization similar to PIN2 (Figs 2a–c, S5), suggesting that the HL harbors molecular cues for the ER vs PM subcellular localization. Additionally, similar to PIN2, the hybrid PIN X1 showed the predominantly apical subcellular localization, as manifested by colocalization of X1‐GFP and PIN2‐mCherry in root epidermal cells (Fig. S5a–c). Consequently, X1 was also able to partially rescue the defective root gravitropism phenotype of the *pin2* mutant (Fig. 2c).

Next, we swapped the N‐TMD of PIN2 with that of PIN6 to make a chimeric protein that we called X2. Notably, as compared to the X1 chimera with both N‐ and C‐TMDs from PIN6, the chimeric X2 protein has much greater domain similarity to PIN2; nonetheless, X2 showed increased localization at the lateral sides of the root epidermal cell PM, as shown by coexpressing it with PIN2‐mCherry, and accordingly failed to rescue the defective root gravitropism of *pin2* (Figs 2d, S5d). Unexpectedly, in root epidermal cells, the chimeric PIN X3, in which we substituted the C‐TMD of PIN2 with that of PIN6, was predominantly localized to the ER, as confirmed by its coexpression analysis with PIN2‐mCherry and the ER marker *HDEL‐RFP*, which was different from the subcellular localization of PIN2, X1 and PIN6 (Figs 2e,g–i, S5e). Consequently, the ER‐localized X3 protein also failed to complement the *pin2* mutant phenotype (Fig. 2e,f). Together, these findings imply that besides the HL, the pairwise matching of N‐ and C‐terminal TMDs contributes to the subcellular localization of PIN proteins. Consistent with this idea, compared with the PM localization of X1 and X2, the chimeric PIN protein X3 was also localized to the ER in LRC/cortex cells similar to its subcellular localization in root epidermis (Fig. S6). Notably, while the expression of ER‐localized X3 under control of the *PIN2* promoter in the wild‐type background showed normal gravitropic root growth (Fig. 2j), expression of nonpolar PIN6 might interfere with the apically PM‐localized PIN2 function and therefore affect normal root gravitropism.

Overall, these results suggest that while the PIN hydrophilic loop contains the main sequence‐based determinants of ER vs PM localization, the N‐ and C‐terminal *trans*‐membrane domains also need to be mutually matched to allow correct PIN subcellular localization.

### Crucial role of pairwise matching between the N‐ and C‐TMDs in PIN subcellular localization

**Fig. 3:**
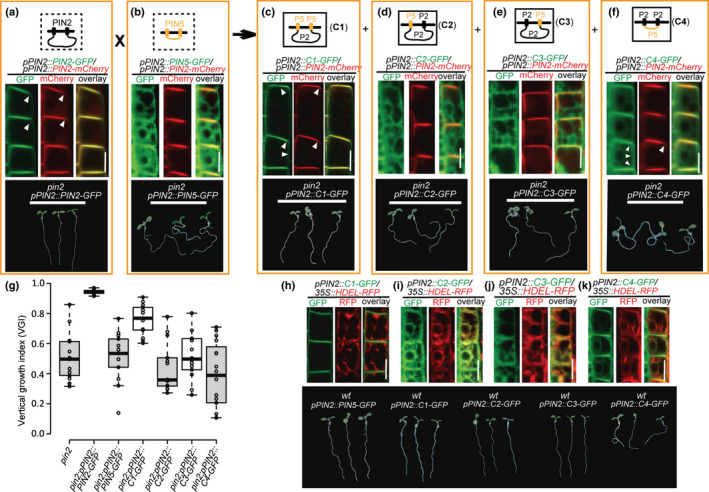
The sequence‐divergent transmembrane domains (TMDs) contribute to the subcellular localization of PIN proteins to the PM or ER. (a–f) Top panels: structural diagrams of PIN2, PIN5 and the chimeric PIN proteins (C1, C2, C3 and C4) with the domains swapped between a canonical PIN (PIN2) and a noncanonical PIN (PIN5). Middle panels: subcellular localization analyses of the PINs in root epidermal cells by their coexpression with the apically PM‐localized PIN2‐mCherry. The chimeric PIN, C1, which consists of the C‐ and N‐TMDs from PIN5 and the hydrophilic loop (HL) domain from PIN2, is localized to the PM as shown by its coexpression with PIN2‐mCherry (c), while the chimeric C2 protein, with the N‐TMD from PIN5 and the C‐TMD and HL from PIN2, and C3, with the C‐TMD from PIN5 and the N‐TMD and HL from PIN2, are mainly localized to the ER (b, d, e). Unlike both PIN2 and PIN5, the chimeric C4 protein, which has the C‐ and N‐TMDs from PIN2 and the HL domain from PIN5, is localized to both the PM and the ER (f). Bottom panels: the results of genetic complementation experiments indicating that C2, C3 and C4 failed to complement the *pin2* mutant phenotype (d–f). The white arrowheads (b, c, f) indicate the direction that the PIN proteins localize to. (g) Quantification of the vertical growth index (VGI) of *PIN* transformants in the *pin2* mutant background (a–e). Center lines show the medians; box limits indicate the 25^th^ and 75^th^ percentiles as determined by R software; whiskers extend 1.5 times the interquartile range from the 25^th^ and 75^th^ percentiles, and outliers are represented by dots. (h–k) Colocalization analysis of the GFP‐fused chimeric PIN proteins C1, C2, C3 and C4 along with the ER marker HDEL‐RFP. (l) Root gravitropic phenotypes of *PIN* gene transformants in the wild‐type background. Expression of C4 driven by the *PIN2* promoter results in defective root gravitropism, while plants expressing the ER‐localized chimeric PINs (C2 and C4) show normal gravitropic root growth. Scale bars, 10 µm.

Next, we performed further domain swapping experiments between PM‐localized PIN2 and ER‐localized PIN5 (Fig. 3a,b). When the short central HL of PIN5 was replaced by the long HL from PIN2, the chimeric PIN protein, called C1, displayed PM localization, further demonstrating the crucial role of the HL domain in subcellular localization of the PIN proteins (Fig. 3c). The colocalization of C1‐GFP with PIN2‐mCherry confirmed its apical localization at the PM, although C1 also shows more lateral localization in epidermal cells as compared to PIN2. Moreover, C1‐GFP partially restores the *pin2* mutant defects in root gravitropism (Figs 3c, S7a–c).

To further test the possible role of the pairwise matched N‐ and C‐TMDs in the subcellular localization of PIN proteins, we replaced either the N‐ or the C‐TMD of PIN2 individually with the corresponding part of PIN5, to generate the chimeric proteins called C2 and C3, respectively (Fig. 3d,e). When compared to C1, the C2 and C3 chimeras shared many more common domains with PIN2; nonetheless, unexpectedly, unlike PIN2 and C1, both C2 and C3 were found to be predominantly localized to the ER but not to the PM in epidermal cells, as indicated by colocalization analysis with PIN2‐mCherry (Figs 3a–e, S7a–e) and *HDEL‐RFP* (Fig. 3h–j). Likewise, in the LRC/cortex cell types, both the chimeric PIN proteins C2 and C3 were localized to the ER while the C1 was predominantly localized to the PM (Fig. S8). Consistent with their ER localization pattern, both C2 and C3 failed to rescue the gravitropic defect of the *pin2* mutant (Fig. 3a,d–e). These results support the suggestion that the mutual matching of TMDs between the N‐ and C‐termini is crucial for the correct subcellular localization of PIN proteins, and also strongly implies that the PIN proteins underwent an intramolecular domain–domain coevolution to ensure compatibility between the domains and thus jointly determine the subcellular membrane localization of PIN proteins.

Furthermore, to evaluate the roles of sequence variation of TMDs between the canonical and noncanonical PINs in subcellular localization, we replaced both N‐ and C‐TMDs of PIN5 with those of PIN2 to create a chimeric protein called C4. Unexpectedly, in contrast to the predominantly ER‐localized PIN5 (Fig. 3b), C4, with the short HL of PIN5, showed a dual localization to both the PM and the ER, as indicated by its colocalization with both PIN2‐mCherry and HDEL‐RFP (Fig. 3f,k). This suggests that not only the HL, as previously shown (Figs 2c, 3c), but also the sequence variation in the TMDs from different clades of PIN proteins contributes to their differential subcellular localizations. Consistent with the nonpolar distribution of the C4 protein, which localizes to the PM (Fig. S7f), expressing C4 in the wild type background under control of the *PIN2* promoter probably interferes with the apically PM‐localized endogenous PIN2 function and therefore leads to the observed defect in root gravitropism (Fig. 3l). By contrast, the transgenic lines expressing the ER‐localized PIN5 and chimeric PIN proteins (C2 and C3) in wild‐type background driven by the *PIN2* promoter showed normal gravitropic root growth (Fig. 3l).

Thus, again PM‐localized but not ER‐localized PIN proteins can interfere with the function of PM‐based endogenous PIN proteins. It also confirms that the sequence variation of TMDs and the compatibility between N‐ and C‐terminal TMDs collectively convey the molecular cues of subcellular localization for PIN proteins.

### Unmatched N‐ and C‐TMDs between PIN5 and PIN6

As shown above, the N‐ and C‐TMDs of PIN6 and PIN5 do not match the counterpart TMDs of PIN2, leading to the retention of X3, C2 and C3 in the ER. We therefore postulated that after the evolutionary divergence of different PIN clades (Fig. 1a), intramolecular domain–domain coevolution occurred independently within every clade, resulting in unmatched N‐ and C‐TMDs, including between PIN5 and PIN6 clades as well.

**Fig. 4:**
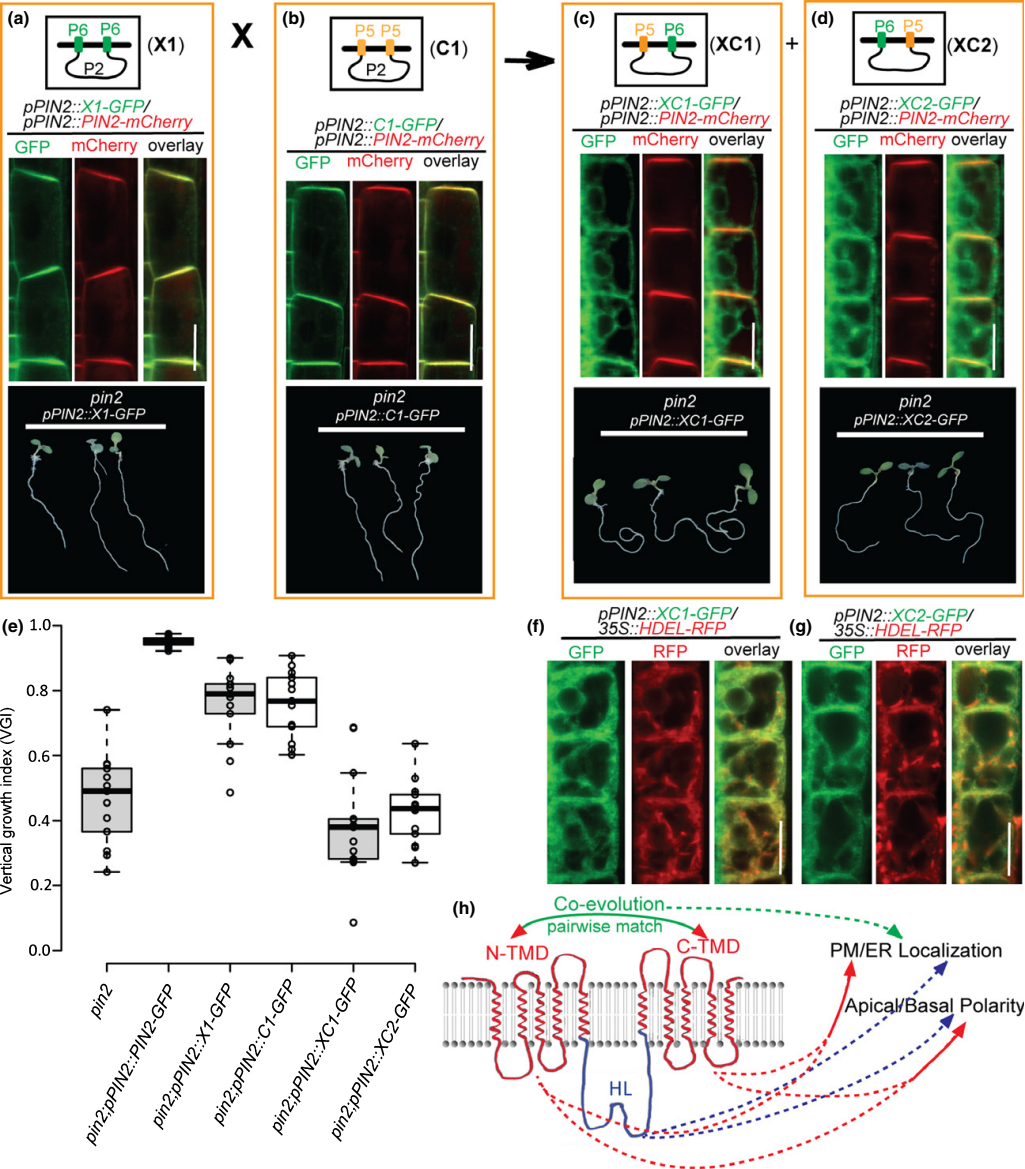
The crucial role of the pairwise‐matched N‐ and C‐terminal transmembrane domains (TMDs) in determining the subcellular localization of PIN proteins. (a–d) Top panels: designs of the chimeric PINs XC1 and XC2 that were constructed by swapping the domains between X1 and C1. Middle panels: colocalization analyses of the chimeric PINs with the apically PM‐localized PIN2‐mCherry protein in root epidermal cells. X1 and C1 are PM‐localized (a, b), whereas XC1 and XC2 are predominantly localized to the ER (c, d). Bottom panels: the results of genetic complementation experiments indicating that the ER‐localized XC1 and XC2 proteins failed to complement *pin2* defective root gravitropism when expressed by the *PIN2* promoter (c, d). (e) Quantification of the vertical growth index (VGI) of the *PIN* transformants in the *pin2* mutant background (a–d). Center lines show the medians; box limits indicate the 25^th^ and 75^th^ percentiles as determined by R software; whiskers extend 1.5 times the interquartile range from the 25^th^ and 75^th^ percentiles, and outliers are represented by dots. (f, g) Colocalization analyses of chimeric proteins XC1 and XC2 with the ER marker HDEL‐RFP. (h) Schematic diagram depicting the roles that the PIN protein domains play in subcellular localization (PM vs ER) and cellular polarity (apical vs basal). In addition to the sequence divergence of the TMD and hydrophilic loop (HL) domains among these PIN clades, we highlight the crucial role of the pairwise matching between the N‐ and C‐TMDs in subcellular localization, which strongly suggests independent intramolecular coevolution of PIN proteins after evolutionary divergence of the PIN clades. Scale bars, 10 µm.

To test this hypothesis, we further swapped the TMDs between PM‐localized X1 and C1, whose N‐/C‐terminal TMDs came from PIN6 and PIN5, respectively (Fig. 4a,b). Two hybrid PIN proteins were then constructed, named XC1 and XC2, which had one terminal TMD derived from PIN5 and the other from PIN6 (Fig. 3c,d). Both XC1 and XC2 completely failed to rescue the *pin2* phenotype, and showed a predominantly ER subcellular localization in epidermal cells, as indicated by coexpressing them with PIN2‐mCherry or HDEL‐RFP (Fig. 4c–g). Moreover, analyzing the subcellular localization of the XC1 and XC2 in cell files of LRC and cortex further confirmed the prominent ER localization of the two chimeric PIN proteins (Fig. S8g,h). Additionally, wild‐type plants expressing XC1 and XC2 under control of the *PIN2* promoter showed normal root gravitropic growth, consistent with the ER‐subcellular localization of these chimeric proteins (Fig. S9).

These results show that not only are the N‐ and C‐TMDs between PIN2 and PIN5 or PIN6 not pairwise matched, but also the TMDs between PIN5 and PIN6 do not match, suggesting that after the PIN5 and PIN6 clades diverged (Fig. 1a), the TMDs of these two clades of PIN proteins also underwent independent evolution, leading to the uncoupling of pairwise matched TMDs between PIN5 and PIN6.

Finally, to further confirm the intramolecular coevolution of PIN protein, we performed intraprotein coevolutionary analysis using several PIN members from various plant species. Bioinformatics analysis identified a multitude of pairs of coevolving sites (11 pairs of amino acids in total) at the correlated regions of N‐ and C‐TMDs (Table S2), suggesting strongly that the intramolecular coevolution of N‐ and C‐transmembrane domains of PIN auxin transporter occurred during PIN evolution, which is consistent with our experimental results. Additionally, it also showed clearly that for most pairs of identified coevolving sites, the two correlated amino acids, separately located at the N‐ and C‐TMDs, were simultaneously substituted/mutated among three different PIN clades (i.e. PIN2, PIN6 and PIN5) (Table S2). The correlated mutations/substitutions of the coevolving sites imply the important role of the pairwise matching of N‐ and C‐terminal TMDs for localization and function of PIN proteins.

## Discussion

PIN protein family members, which comprise the crucial polar auxin transporters, are ubiquitous across the plant kingdom and evolved to be localized to diverse membrane compartments including the ER and the different polar domains at the PM. There is a long‐standing question as to why the PIN protein family members with similar topological structures evolved to localize to different cellular compartments and thus flexibly transport auxin within or between cells.

Our work provided initial insights into how the variation in different domains of PIN proteins contribute to their differential membrane localization (Fig. 4h): the central hydrophilic loops of PIN proteins are the main determinants of PIN’s subcellular localization, as evidenced by the fact that the ER‐localized PIN6 and PIN5 proteins are targeted to the PM after swapping their HLs with that of PM‐localized PIN2; the transmembrane domain modules also contribute to the subcellular localization, as replacing the N‐ and C‐TMDs of the predominantly ER‐localized PIN5 with those of PIN2 results in the dual localization of the chimeric PIN protein C4 to both the PM and the ER (Fig. 3f); and the pairwise matching between the N‐ and C‐TMDs also plays a crucial role in determining the subcellular localization, which is evidenced by the predominantly ER localization of a series of chimeric PINs combining the ‘long’ HL domain of the PM‐localized PIN2 with the hybrid N‐ and C‐TMDs from two different PIN clades. This points to the intramolecular domain–domain coevolution after the divergence of the PIN protein clades. In the future, revealing the PIN structure would be very helpful in understanding the underlying mechanism of PIN subcellular localization determined by pairwise matched N‐ and C‐TMDs. Additionally, TMDs exist ubiquitously in membrane proteins, so an outstanding question remains whether the coevolution and matching of the TMDs are more commonly implicated in determining the subcellular localization and function of other membrane proteins.

Our systematic domain swapping experiments among the PIN family members not only elucidated the divergent PM/ER localization of the PIN proteins, but also provided insights into how the polar PM localization (apical vs basal) is determined by the domain variation in PIN proteins (Fig. 4h). The HL plays an important role in the polar localization of the canonical PIN, as shown by the intergenic swapping of the HL domain between PIN2 and PIN1 (Fig. 1). Interestingly, replacing the HL domain of the ancestral form of the canonical PIN, *Marchantia polymorpha* PINZ (MpPINZ), with the HL of *Arabidopsis* PIN2, caused its polarity to switch from the basal side to the apical side in epidermal cell PM, and therefore successfully restored the defective root gravitropism of the *pin2* mutant (Bowman *et al*., 2017; Zhang *et al*., 2019). These findings imply strongly that the HL domain of the canonical PIN family members experienced evolutionary selection, and therefore led to their divergent cellular localizations and functions during plant evolution. Furthermore, the TMDs also contribute to the polar localization of PIN proteins; replacement of the TMDs of PIN2 with those of PIN5 resulted in enhanced lateral localization when compared with the original PIN2 protein (Figs 3c, S7c).

In the green alga *Klebsormidum*, only one *PIN* gene (*KfPIN*) has been identified, and the protein it encodes is PM‐localized and also transports auxin with high specificity (Skokan *et al*., 2019). In the representatives of early diverging land plants, *Marchantia* and other bryophytes, the PIN proteins have diverged into two clades: the canonical PINs localized in the PM (e.g. *Physcomitrella* PINA/B/C), and noncanonical PINs localized to the ER (e.g. *Physcomitrella* PIND) (Viaene *et al*., 2014). In the higher plant *Arabidopsis*, however, in addition to these two PIN clades, there is another particular PIN protein, PIN6, that shows dual localization to both the PM and the ER (Simon *et al*., 2016). The different localization between chimeric X2 protein (PM‐localized) and X3 protein (ER‐localized) implies that the N‐TMD of PIN6 is able to partially match with the C‐TMD of PIN2 (Fig. 2d), while the C‐TMD of PIN6 is fully unmatched with the C‐TMD of PIN2 (Fig. 2e). However, the C2 and C3 chimeras showed the same subcellular localization at the ER (Fig. 3d,e), indicating that the N‐ and C‐TMDs between PIN2 and PIN5 are mutually unmatched. These results suggest strongly that, despite the fact that PIN6 is a noncanonical PIN, it probably shares much closer common descent with canonical PINs such as PIN2, rather than the ER‐localized noncanonical PIN proteins (e.g. PIN5), which is further supported by our domain swapping results that the TMDs of PIN5 and PIN6 are fully unmatched, leading to the ER localization of these chimeric PIN proteins XC1 and XC2 (Fig. 4c,d). Moreover, in contrast to the PM‐localized X1, the chimera X2, localized to the PM, failed to similarly partly revert the *pin2* agravitropic root phenotype (Fig. 2c,d), suggesting that pairwise matching of N‐ and C‐TMDs might contribute to PIN auxin transport activity, in addition to the contribution to the subcellular localization of PIN proteins.

Recently, evidence has accumulated to demonstrate the existence of intramolecular coevolution within proteins and intermolecular coevolution among interacting proteins. Specifically, changes in the amino acid residues of one protein domain or one protein would impose evolutionary pressure on the other domains or proteins with which it interacts, thus resulting in corresponding residue changes in order to maintain or re‐establish the domain–domain or protein–protein interaction (Yeang & Haussler, 2007; Lynch & Hagner, 2015; Cong *et al*., 2019). Here, our results imply that the intramolecular coevolution also took place in PIN proteins, providing additional insight into the evolution of membrane proteins.

It has been proposed that the differential cellular polarity and subcellular localization of PIN proteins relies on cargo recognition during protein sorting (Adamowski & Friml, 2015; Zwiewka *et al*., 2019). However, it remains to be addressed whether the subcellular localization determined by this pairwise matching of N‐/C‐TMDs results from such failed cargo recognition processes or could be due to protein misfolding, given that the misfolded protein may fail to be sorted to the PM and therefore be retained in the ER.

## Author contributions

YZ and JF conceived the research and designed the experiments. YZ and CH performed the experiments. XW performed the bioinformatics analysis for the intraprotein coevolution of PINs. YZ and JF wrote the manuscript. All authors contributed to the manuscript and discussed the results extensively.

## Supporting information


**Dataset S1** Amino acid sequence alignment of the PIN proteins from 15 plant species detailed in the Materials and Methods section regarding the identification of coevolving sites.
**Fig. S1** Differential subcellular localizations of three PIN protein clades in *Arabidopsis*.
**Fig. S2** Polar localization of the chimeric PIN proteins in which the domains were swapped between the PM‐localized canonical PINs, PIN1 and PIN2.
**Fig. S3** Subcellular localization of the chimeric PIN proteins in cells of the LRC and cortex.
**Fig. S4** Loss of root gravitropism in the *Arabidopsis pin2* mutant.
**Fig. S5** Subcellular localizations of chimeric PIN proteins with domains swapped between PIN2 and the noncanonical PIN6.
**Fig. S6** Subcellular localization of the chimeric PIN proteins in cells of the LRC and cortex.
**Fig. S7** Subcellular localizations of chimeric PINs with domains swapped between PIN2 and the noncanonical PIN5.
**Fig. S8** Subcellular localization of the chimeric PIN proteins in cells of the LRC and cortex.
**Fig. S9** Root gravitropic analysis of *Arabidopsis* seedlings expressing ER‐localized chimeric PINs in the wild‐type background.
**Table S1** Primers used for vector construction.
**Table S2** Identification of coevolving sites at the correlated N‐ and C‐TMDs of PIN proteins.
Please note: Wiley Blackwell are not responsible for the content or functionality of any Supporting Information supplied by the authors. Any queries (other than missing material) should be directed to the *New Phytologist* Central Office.
