# Supplementary material for: Directional auxin fluxes in plants by intramolecular domain–domain coevolution of PIN auxin transporters
Source: New Phytol. 2020 May 20;227(5):1406–16. doi: 10.1111/nph.16629 (PMC7496279; doi:10.1111/nph.16629)
Supplement: Supplementary file 1 — Dataset S1 Amino acid sequence alignment of the PIN proteins from 15 plant species detailed in the Materials and Methods section regarding the identification of coevolving sites. Fig. S1 Differential subcellular localizations of three PIN protein clades in Arabidopsis. Fig. S2 Polar localization of the chimeric PIN proteins in which the domains were swapped between the PM‐localized canonical PINs, PIN1 and PIN2. Fig. S3 Subcellular localization of the chimeric PIN proteins in cells of the LRC and cortex. Fig. S4 Loss of root gravitropism in the Arabidopsis pin2 mutant. Fig. S5 Subcellular localizations of chimeric PIN proteins with domains swapped between PIN2 and the noncanonical PIN6. Fig. S6 Subcellular localization of the chimeric PIN proteins in cells of the LRC and cortex. Fig. S7 Subcellular localizations of chimeric PINs with domains swapped between PIN2 and the noncanonical PIN5. Fig. S8 Subcellular localization of the chimeric PIN proteins in cells of the LRC and cortex. Fig. S9 Root gravitropic analysis of Arabidopsis seedlings expressing ER‐localized chimeric PINs in the wild‐type background. Table S1 Primers used for vector construction. Table S2 Identification of coevolving sites at the correlated N‐ and C‐TMDs of PIN proteins. Please note: Wiley Blackwell are not responsible for the content or functionality of any Supporting Information supplied by the authors. Any queries (other than missing material) should be directed to the New Phytologist Central Office. [file NPH-227-1406-s001.zip › nph16629-sup-0001-FigsS1-S9 -TablesS1- S2.pdf]

New Phytologist

***New Phytologist* Supporting Information**

Article title: **Directional auxin fluxes in plants by intramolecular domain-domain co-evolution of PIN auxin transporters**

Authors: Yuzhou Zhang[1], Corinna Hartinger[1], Xiaojuan Wang[2] and Jiří Friml[1]

Article acceptance date: 12 April 2020

The following Supporting Information is available for this article:

Fig. S1 Differential subcellular localizations of three PIN protein clades in *Arabidopsis*. a-d, Epidermal cells from the transition zone and meristem zone of *Arabidopsis* roots. PIN2-GFP (**a**) and PIN1-GFP (**b)** are localized at the plasma membrane (PM), but are separately localized to the apical or basal sides of the epidermal cells, indicated by the white arrows. PIN6-GFP is simultaneously localized to the PM and the endoplasmic reticulum (ER) (**c**), whereas PIN5 is predominantly ER-localized (**d**). Scale bars, 10 μm.

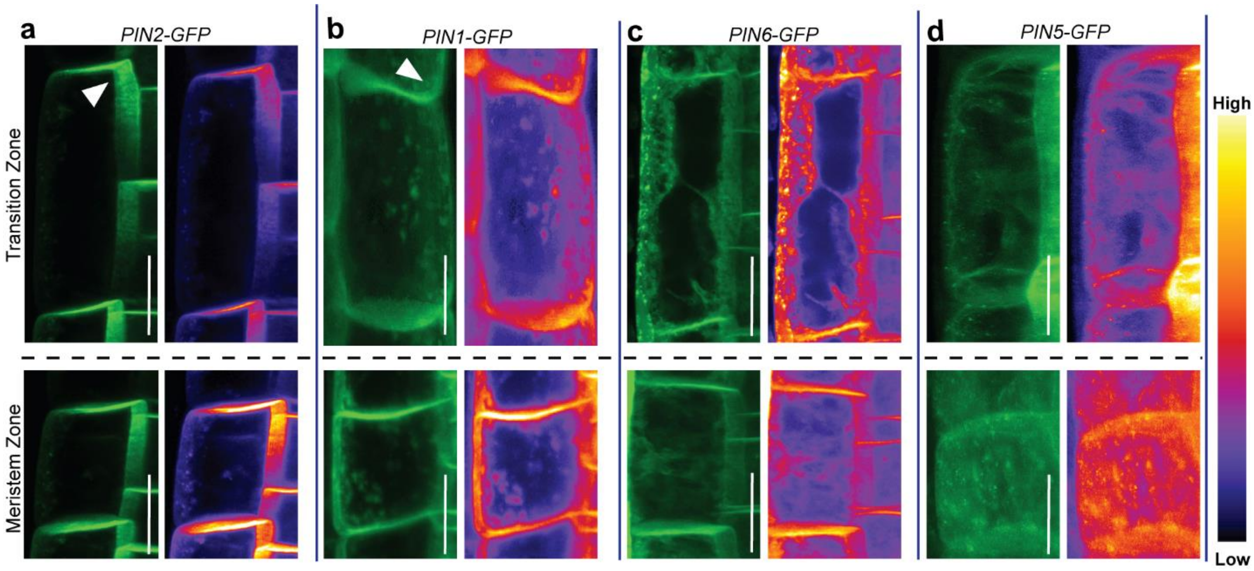

Fig. S2 Polar localization of the chimeric PIN proteins in which the domains were swapped between the PM-localized canonical PINs, PIN1 and PIN2. a-f, Co-localization analyses of the chimeric PIN proteins in *Arabidopsis* root epidermal cells by co-expressing them under control of *PIN2* promoter with apically PM-localized PIN2-mCherrry. The white arrowheads indicate the direction that the PIN proteins localize to. Scale bars, 10 μm.

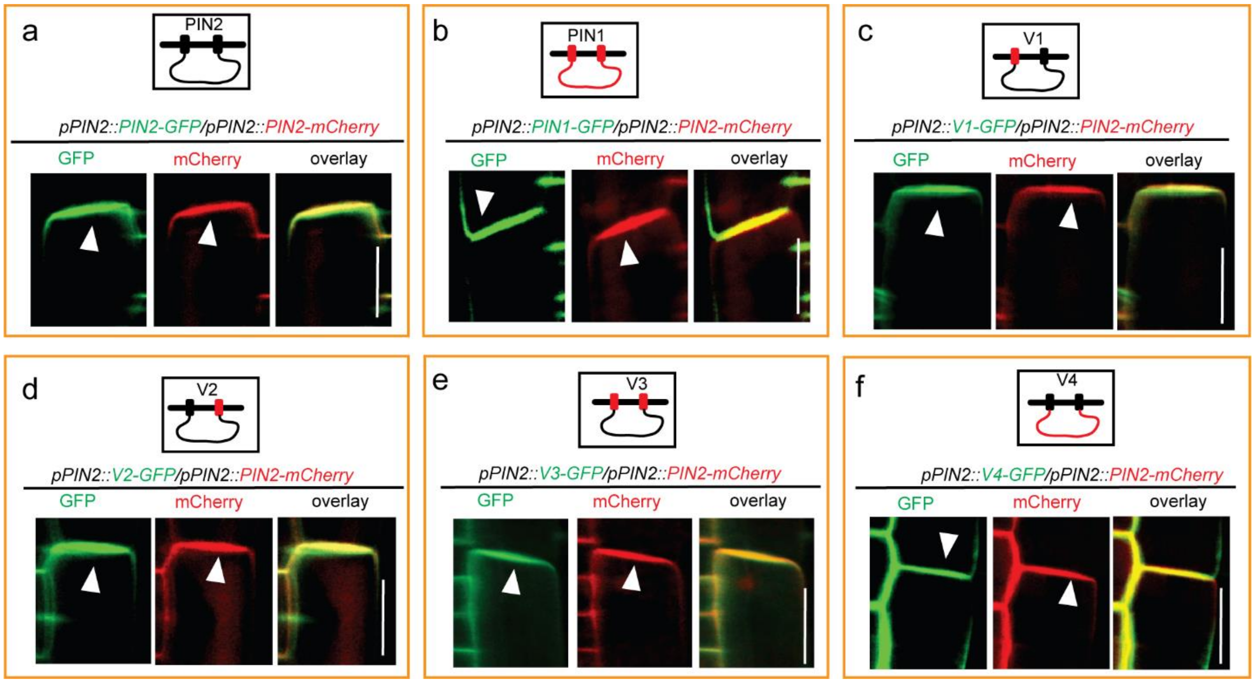

Fig. S3 Subcellular localization of the chimeric PIN proteins in the cells of LRC and cortex.

a-f, Subcellular localization analyses of the chimeric PIN proteins with the domains swapped between PM-localized PIN1 and PIN2 in *Arabidopsis* cells of lateral root cap (LRC) and cortex. The yellow arrowheads indicate the direction that the PIN proteins localize to. Scale bars, 10 μm.

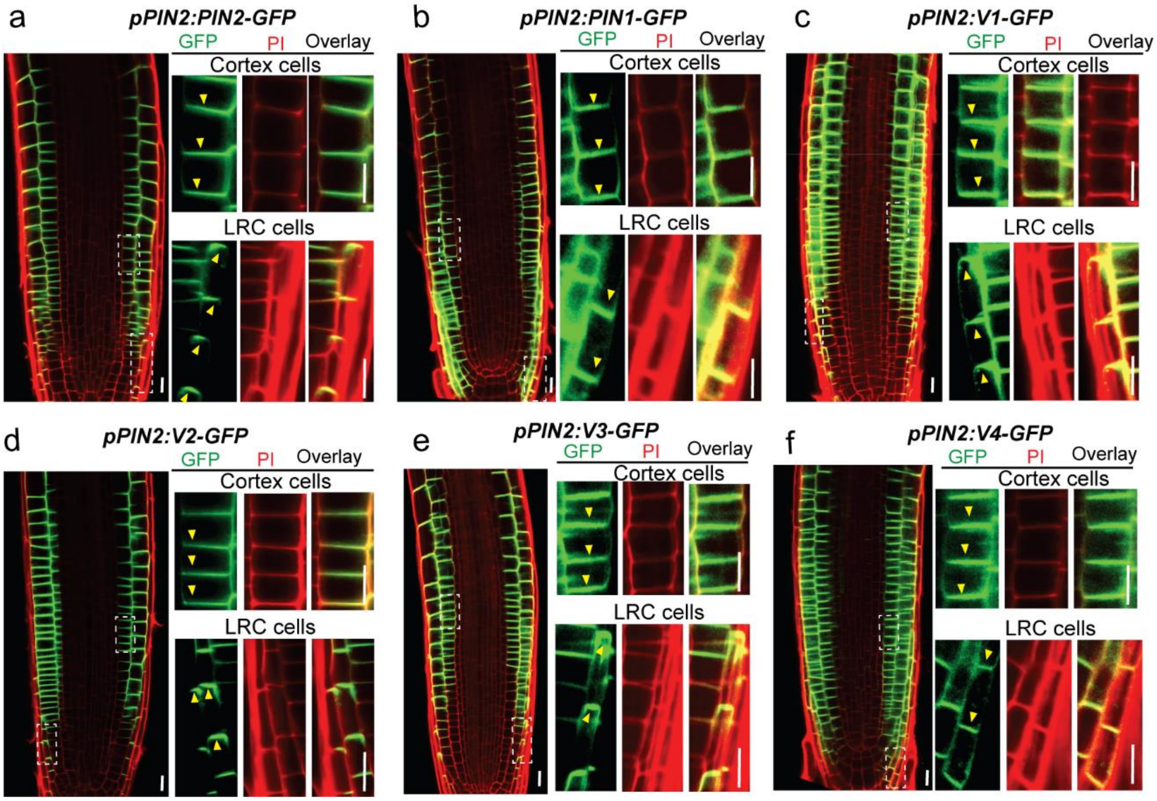

Fig. S4 Loss of root gravitropism in the *Arabidopsis pin2* mutant. a, When compared with wild type, the *pin2* mutant shows defective root gravitropism. **b**, Diagram showing the vertical growth index (VGI) used in the measurement of root gravitropism (full length of root (L) divided by vertical distance travelled (L_y)).

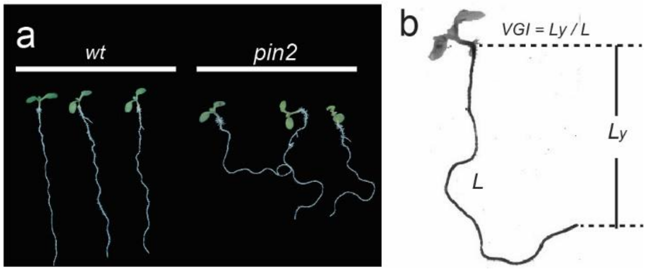

Fig. S5 Subcellular localizations of chimeric PIN proteins with domains swapped between PIN2 and the non-canonical PIN6. a-e, Co-localization analyses of the GFP-fused chimeric PIN proteins X1, X2, and X3 with the apically PM-localized PIN2-mCherrry as shown in Fig. 2 after co-expressing them in *Arabidopsis* root epidermal cells. The white arrowheads indicate the direction that the PIN proteins localize to. Scale bars, 10 μm.

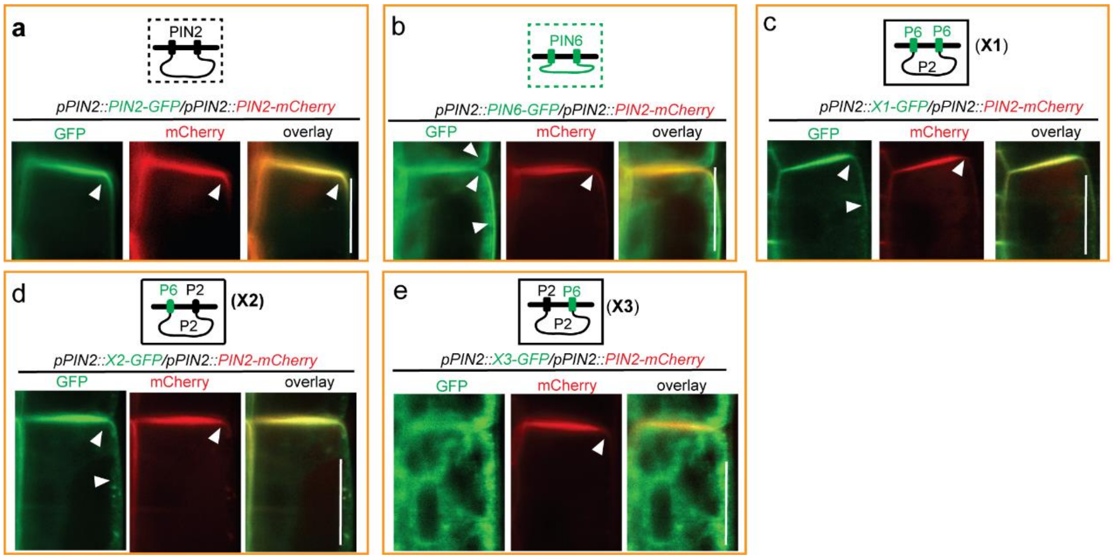

New Phytologist

Fig. S6 Subcellular localization of the chimeric PIN proteins in the cells of LRC and cortex.

a-e, Subcellular localization analyses of the chimeric PIN proteins with the domains swapped between canonical PIN2 and non-canonical PIN6 in *Arabidopsis* cells of lateral root cap and cortex. The yellow arrowheads indicate the direction that the PIN proteins localize to. Scale bars, 10 µm.

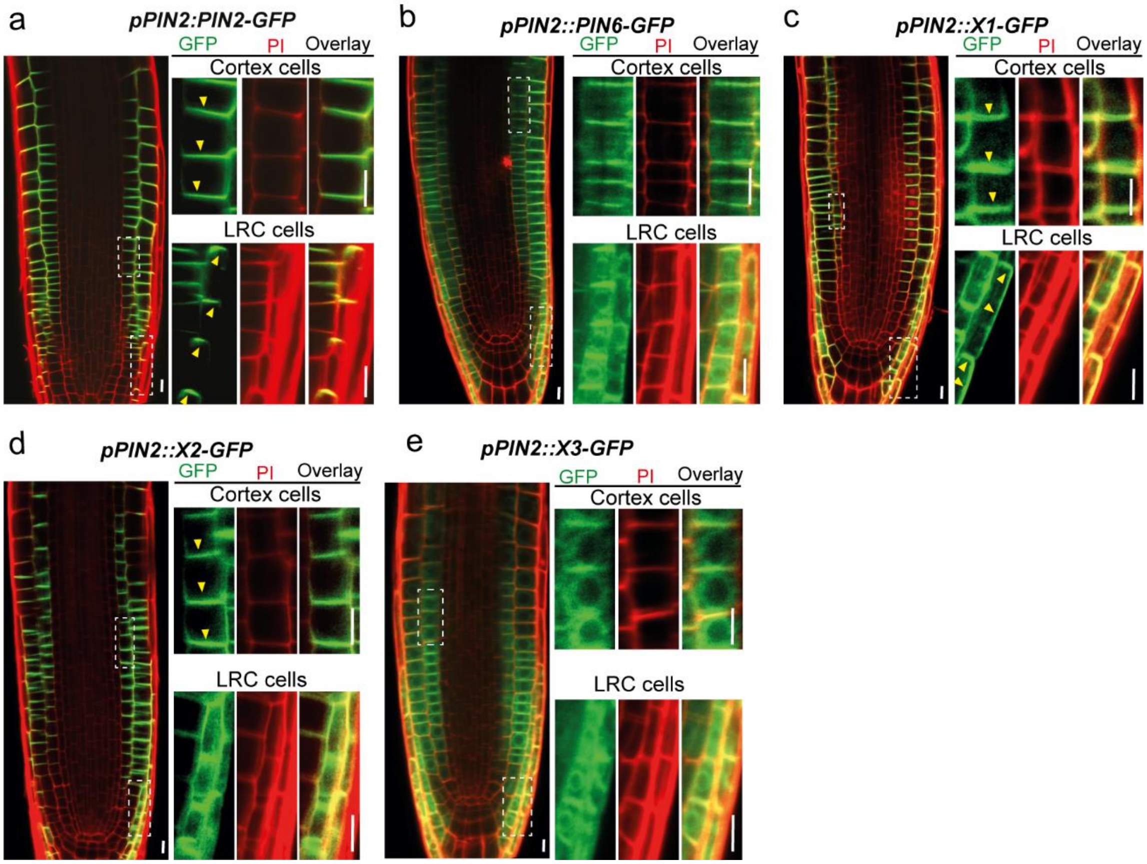

New Phytologist

Fig. S7 Subcellular localizations of chimeric PINs with domains swapped between PIN2 and the non-canonical PIN5. a-f, Co-localization analyses of the GFP-fused chimeric PIN proteins C1, C2, C3, and C4 with the apically PM-localized PIN2-mCherrry as shown in Fig. 3a-f. The white arrowheads indicate the direction that the PIN proteins localize to. Scale bars, 10 μm.

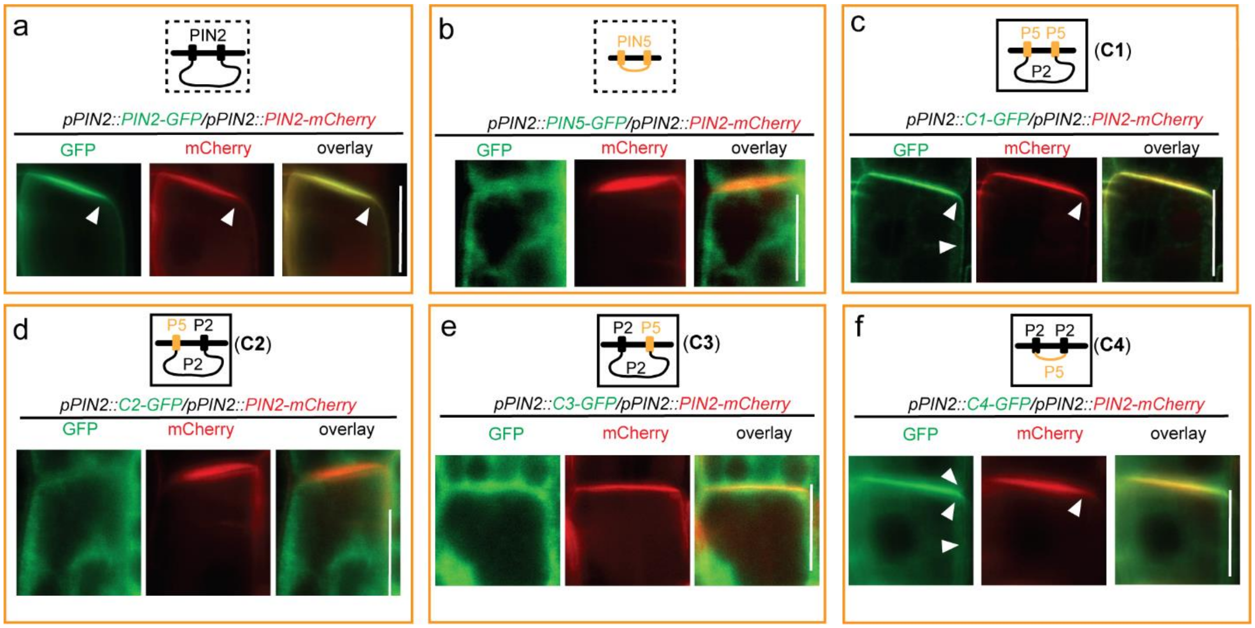

Fig. S8 Subcellular localization of the chimeric PIN proteins in the cells of LRC and cortex.

a-f, Subcellular localization analyses of the chimeric PIN proteins with the domains swapped between PM-localized PIN2 and ER-localized PIN5 in *Arabidopsis* cells of lateral root cap (LRC) and cortex. **g, h,** Subcellular localization analyses of the chimeric PIN proteins with the domains swapped between chimeric PINs, X1 and C1 (shown in Fig. 4), in *Arabidopsis* cells of lateral root cap and cortex. The yellow arrowheads indicate the direction that the PIN proteins localize to. Scale bars, 10 μm.

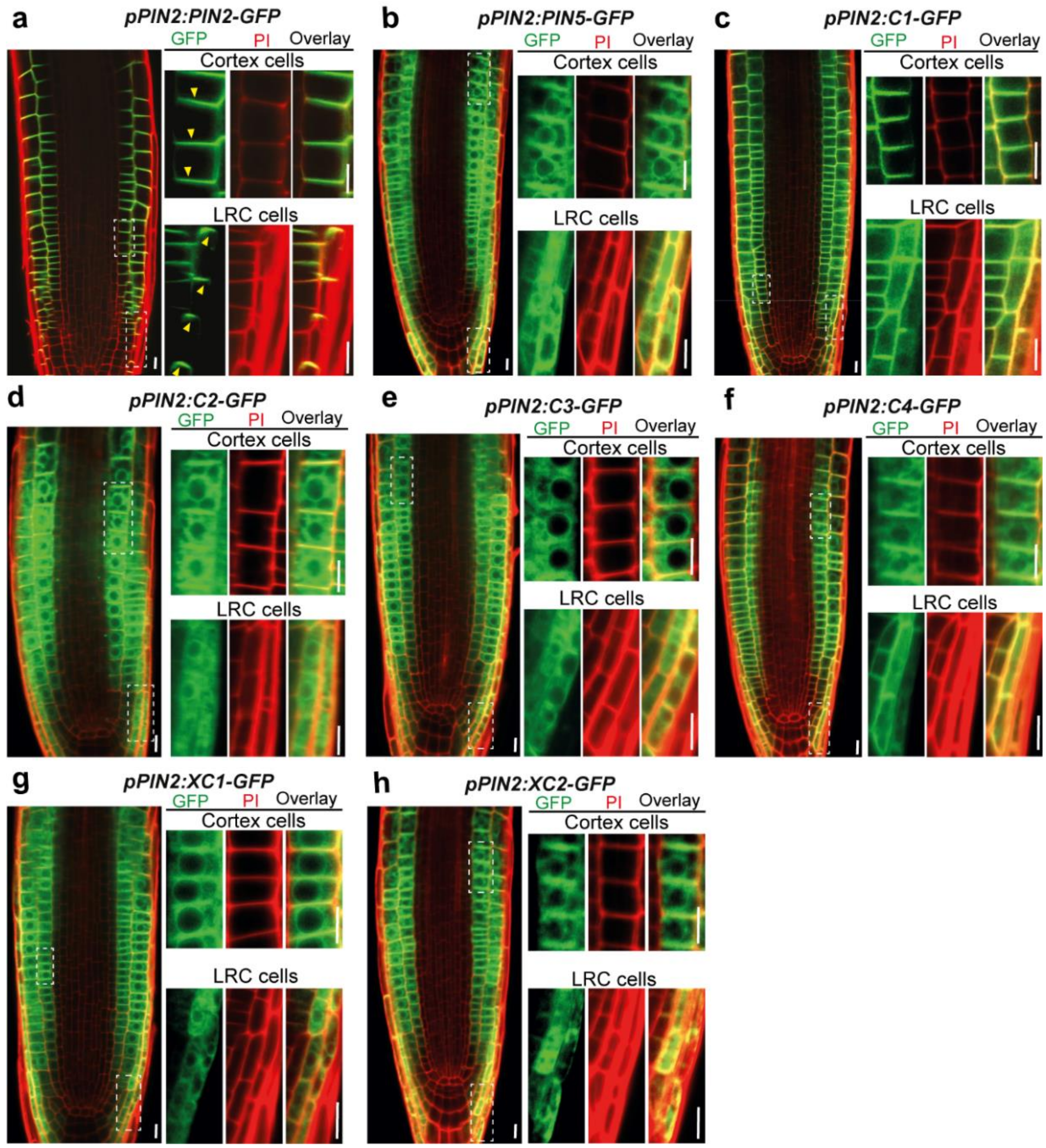

Fig. S9 Root gravitropic analysis of *Arabidopsis* seedlings expressing ER-localized chimeric PINs in the wild-type background. The transgenic seedlings expressing the chimeric PIN proteins, XC1 or XC2, by *PIN2* promoter showed normal root gravitropic growth.

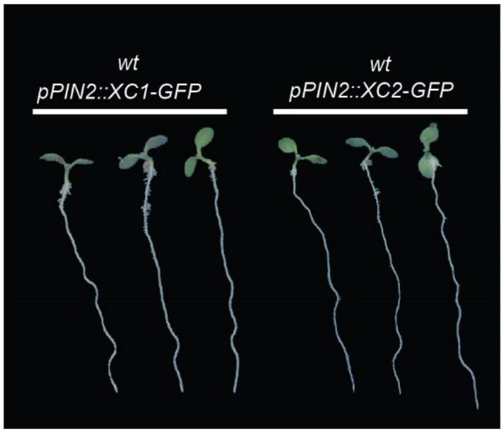

New Phytologist

Table S1 Primers used for vector construction

Gene	Primer sequences
Primer sequences used for the construction of chimeric PIN by overlapping PCR	
PIN2 promoter	5′- TATAGAAAAGTTGTAAATAGTTTCATCCTGTTTTATCAGGCTACATTCAC -3′<br>5′- TTTGTACAAACTTGATTTGATTTACTTTTTCCGGCGAGAGAGAGAAGAAG -3′
PIN2 N-TMD	5′- GGGGACAAGTTTGTACAAAAAAGCAGGCTTCATGATCACCGGCAAAGAC -3′<br>5′- AACTGCTCGGAGATCAAAAGCTTAGCCCCACGGAACTC -3′
PIN2 C-TMD	5′- CAAGACTCATTCTCATTATGGTTTGGAGAAAACTCATT -3′<br>5′- GGGGACCACTTTGTACAAGAAAGCTGGGTTTTAAAGCCCCAAAAGAAC -3′
PIN1 HL	5′- CTTTTGATCTCCGAGCAG -3′<br>5′- CATAATGAGAATGAGTCT -3′
PIN1 N-TMD	5′- GGGGACAAGTTTGTACAAAAAAGCAGGCTTCATGATCACCGGCAAAGAC -3′<br>5′- CTTAGCCCCACGGAACTC -3′
PIN1 C-TMD	5′- GTTTGGAGAAAACTCATT -3′<br>5′- GGGGACCACTTTGTACAAGAAAGCTGGGTTTTAAAGCCCCAAAAGAAC -3′
PIN2 HL	5′- GAGTTCCGTGGGGCTAAGCTTTTGATCTCCGAGCAG -3′<br>5′- AATGAGTTTTCTCCAAACAGACTAATTCTGATAATG -3′
PIN6 N-TMD	5′- AAAAAAGCAGGCTTCATGATAACGGGAAACGAA -3′<br>5′- CTGCTCGGAGATGAGAAGCCTCGCTGCTCGGAGTTC -3′
PIN6 C-TMD	5′- AGACTAATTCTGATAATGGTGGGCCGCAAGCTTTCT -3′<br>5′- CAAGAAAGCTGGGTTTCATAGGCCCAAGAGGAC -3′
PIN5 N-TMD	5′- AAAAAAGCAGGCTTCATGATAAATTGTGGAGAT -3′<br>5′- CTGCTCGGAGATGAGAAGTCCGGCTTTTCTAAACTC -3′
PIN5 C-TMD	5′- AGACTAATTCTGATAATGGTTTGGTTGAAGCTTGCA -3′<br>5′- CAAGAAAGCTGGGTTTCAATGAATAAACTCCAG -3′
PIN5 HL	5′- GAGTTCCGTGGGGCTAAGTTTAGTAGTAATAATATC -3′<br>5′- AATGAGTTTTCTCCAAACGAGGGACATGACCTCAAGG -3′

Table S2 Identification of co-evolving sites at the correlated N- and C-TMDs of PIN proteins. Co-evolution analysis of the correlated N- and C-terminal TMDs' sites in PIN proteins across plant species. The pairs of co-evolving sites within PIN molecular were identified by using evolutionary-network models implemented in Spidermonkey/BGM. Posterior probabilities was given for site 1 and site 2 to be not conditionally independent, determined by Spidermonkey/BGM. The results consist of the set of putatively co-evolving sites in the alignment with the posterior probability exceeding a default cut-off of 0.5. Site positions were given in reference to the aligned PIN amino acid sequence, and the amino acid sequence of PIN2, PIN6 and PIN5, respectively (from left to right in the table).

Aligned PIN sequence				PIN2			PIN6			PIN5	
Amino acid position 1 (N-TMD)	Amino acid position 2 (C-TMD)	Posterior probability		Amino acid position 1 (N-TMD)	Amino acid position 2 (C-TMD)		Amino acid position 1 (N-TMD	Amino acid position 2 (C-TMD)		Amino acid position 1 (N-TMD	Amino acid position 2 (C-TMD)
**53**	**870**	**0,99**		**L53**	**K614**		**V53**	**R537**		**L53**	**K316**
**56**	**799**	**0,55**		**F56**	**S546**		**F56**	**S469**		**I56**	**N248**
**78**	**752**	**0,65**		**Q78**	**I499**		**S78**	**S422**		**S78**	**A201**
**78**	**847**	**0,96**		**Q78**	**L593**		**S78**	**R516**		**S78**	**V295**
**78**	**890**	**0,73**		**Q78**	**A634**		**S78**	**S557**		**S78**	**S336**
**83**	**878**	**0,96**		**L83**	**I622**		**F83**	**L545**		**V83**	**V324**
**135**	**795**	**0,54**		**T113**	**M542**		**T113**	**M465**		**S113**	**T244**
**137**	**791**	**0,88**		**V115**	**A538**		**V115**	**A461**		**V115**	**A240**
**152**	**824**	**0,62**		**G130**	**V571**		**Q130**	**I494**		**V130**	**L273**
**152**	**828**	**0,86**		**G130**	**T575**		**Q130**	**S498**		**V130**	**A277**
**152**	**832**	**0,56**		**G130**	**V579**		**Q130**	**F502**		**V130**	**A281**

Dataset. S1 The amino acid sequence alignment of the PIN proteins from 15 plant species showed in the Method section regarding the identification of co-evolving sites.
